# A Smart Contract-Based Dynamic Consent Management System for Personal Data Usage under GDPR

**DOI:** 10.3390/s21237994

**Published:** 2021-11-30

**Authors:** Mpyana Mwamba Merlec, Youn Kyu Lee, Seng-Phil Hong, Hoh Peter In

**Affiliations:** 1 Department of Computer Science and Engineering, Korea University, Seoul 02841, Korea; mlecjm@korea.ac.kr; 2 Department of Computer Engineering, Hongik University, Seoul 04066, Korea; 3 Management Support Division, Hancom WITH, Inc., Pangyo 13493, Korea; sengphil@hancom.com

**Keywords:** blockchain, data privacy and security, dynamic consent management, general data protection regulation (GDPR), smart contract

## Abstract

A massive amount of sensitive personal data is being collected and used by scientists, businesses, and governments. This has led to unprecedented threats to privacy rights and the security of personal data. There are few solutions that empower individuals to provide systematic consent agreements on distinct personal information and control who can collect, access, and use their data for specific purposes and periods. Individuals should be able to delegate consent rights, access consent-related information, and withdraw their given consent at any time. We propose a smart-contract-based dynamic consent management system, backed by blockchain technology, targeting personal data usage under the general data protection regulation. Our user-centric dynamic consent management system allows users to control their personal data collection and consent to its usage throughout the data lifecycle. Transaction history and logs are recorded in a blockchain that provides trusted tamper-proof data provenance, accountability, and traceability. A prototype of our system was designed and implemented to demonstrate its feasibility. The acceptability and reliability of the system were assessed by experimental testing and validation processes. We also analyzed the security and privacy of the system and evaluated its performance.

## 1. Introduction

An enormous amount of sensitive personal data is being collected and used by scientists, businesses, and governments. This has led to unprecedented threats to personal privacy rights and the security of personal data [1,2]. Furthermore, owing to a lack of sufficient transparency, accountability, and user control on personal data usage in traditional centralized systems, personal data has lately been reused for mass surveillance and censorship purposes [3,4]. Data protection authorities are obliged to elucidate the requirements of lawful personal data uses. The European Union (EU) data protection board has published a bill on the need to safeguard personal information [4]. The General Data Protection Regulation (GDPR) mandates institutions, regardless of their location, to have a legal basis to collect and use personal data of EU members’ citizens and residents [5]. Consent is seen as one of the legal foundations for collecting and processing data under the GDPR. However, consent is judged valid only when it is freely given, specific, informed, and unambiguous. Legal guardians must consent on behalf of minors. Individuals are also given the right to change, withdraw, or revoke consent at any time.

### 1.1. Dynamic Consent Management

Dynamic consent management (DCM) is a novel means of engaging individuals in the use of their personal information [6,7,8,9,10,11,12,13,14]. It aims to address the limitations of paper-based and static consent methods, which afford fewer possibilities and less flexibility for people to systematically define and manage their consent preferences. The basic concept of DCM is to give control back to the users to dynamically manage their consent on personal data collection and usage [7,8,9]. However, transparency, accountability, security, and privacy, which are difficult to achieve with traditional dynamic consent management systems (DCMSs) that rely on trusted third parties (TTP), should be sufficiently guaranteed. Information technologies are used to satisfy legal and regulatory requirements for consent agreements and provide personalized interfaces for user interactions. DCMSs have the potential to address inter-sector, cross-border, and large-scale data-sharing challenges to unlock economic benefits and create new business opportunities [10].

A flexible and dynamic consent management approach is required to achieve specific, informed, and engaged consent agreements for legal personal data usage with guarantees of transparency, security, and privacy. However, there are few solutions that systematically empower individuals to (1) provide tailored consent agreements on distinct personal data, and (2) to take control and decide who will collect, access, and/or use their data for specific purposes and periods. In addition, users should be able to delegate consent rights (if necessary or in emergencies when users are unable to provide consent directly), access consent-related information, and withdraw previously given consent at any time. A few recent research works [11,12,13,14,15,16,17] have tried to address some of the aforementioned challenges. However, they are mostly domain-specific and centralized systems that lack trusted data provenance, transparency, and accountability features.

To design a dynamic consent management system for the legal use of personal data under GDPR, the subsequent design requirements (DRs) have to be satisfied:

- DR1: *Key stakeholders and roles identification*–Who are the key stakeholders and what are their legitimate roles and responsibilities?
- DR2: *User-centric dynamic consent management*–How should data subjects be systematically empowered to control their personal data collection and usage consent?
- DR3: *Consent and rights expression*–How can individual consent and rights be explicitly and systematically expressed in a human/machine-readable format?
- DR4: *Consent and data usage activity history accessibility*–How should data subjects be given access to their personal data collection and usage activity history?
- DR5: *Consent violation notification*–How should data subjects be notified, and regulators be informed, about the consent agreement violation?
- DR6: *Consent agreement withdrawal*–What should be done when the consent agreement clauses are violated or the purpose of the acquired consent is no longer valid?

### 1.2. Our Contributions

The key contributions made by this study are summarized as follows:

- We propose a smart-contract-based dynamic consent management system architecture backed by blockchain technology for legal personal data usage based on GDPR.
- We propose user-centric dynamic consent management schemes that leverage smart contracts to enable users to take control of their consent on the personal data collection and usage throughout the data lifecycle. Consent rights can be delegated and audited by users by tracking the log events on the blockchain. Users get notified about any consent agreement clause violation and can withdraw the related consent.
- We integrate decentralized IPFS [18] nodes with our system to store data off-chain. The transactions history and access log data are recorded on the blockchain to enforce trust and provide tamper-proof data provenance, accountability, and traceability.
- A prototype of our system was designed and implemented on top of the Quorum blockchain platform [19] to determine its feasibility. The acceptability and reliability of the system were assessed by experimental testing and validation processes. The implemented code and artifacts have been made publicly available (https://github.com/mlecjm/sc-dcms, accessed on 25 November 2021).
- We present the security and privacy analysis of the system and evaluate its performance in terms of smart contract algorithm complexity, transaction throughput and latency, computing resource usage, and storage network bandwidth utilization.

The rest of this paper is organized as follows. Section 2 elaborates on the background and related work. Section 3 describes the proposed system. Section 4 provides the implementation and experiment details. The system security and privacy analysis, design requirements assessment, and performance evaluation are provided in Section 5. Section 6 discusses the limitations and open challenges. Section 7 concludes the paper and presents the scope for future work.

## 2. Background and Related Work

This section provides background information and describes the state-of-the-art approaches towards dynamic consent management using blockchain technology.

### 2.1. Blockchain and Smart Contract

Blockchain is a type of Distributed Ledger Technology (DLT) initially introduced as a technology underpinning Bitcoin [20]. It records a list of transactions in a chain of blocks that are cryptographically linked and secured. The transaction records in a block are impossible to be modified without retroactively modifying all subsequent blocks in the chain and without the consensus of a majority of network participants. To unlock the potential of blockchain technology, smart contracts (SCs) have been featured by Ethereum [21], which is an open-source, Turing-complete, and general-purpose blockchain platform. SCs are self-sufficient computer programs, which once started, execute automatically and mandatorily the conditions already set (i.e., the agreement or negotiation of a contract) [21]. SCs run accurately as programmed with no possibility of interruption, censorship, fraud, or third-party interference. The blockchain can be divided into two main categories: permissionless and permissioned blockchain. Permissioned blockchains require prior authorization, whereas permissionless blockchains allow anyone to join and participate in the system operations [22]. Based on governance approaches, blockchain networks can be classified into three types of networks: public, private, and consortium networks [22].

- Public blockchain networks are permissionless blockchain networks that enable parties to transact securely in trustless environments. In public blockchain networks such as Bitcoin [20] and Ethereum [21], everyone can join the network anonymously, and all participants having a copy of the ledger can create, verify, and validate transactions.
- Private blockchain networks are permissioned blockchain networks restricted to well-known and previously registered participants of an organization, which are authorized to access and use the network. Compared to public blockchain networks, private blockchain networks are more scalable and take less time for the network to reach a consensus, resulting in faster transactions. However, it is argued that they are not truly decentralized.
- Consortium blockchain networks, also known as hybrid or federated blockchain networks, are semi-decentralized blockchain networks, which combine public and private blockchain features. A consortium blockchain network is open only to a selected group of organizations or individuals that have decided to share the ledger among themselves for transactions. Some examples are R3 Corda and Quorum.

Table 1 presents a comparison of five popular blockchain platforms [19,21,22,23] considering their key features, which include targeted industry, mode of operation, ledger permission, consortium network, decentralization, transaction and SC privacy, consensus protocols, transaction throughput, blockchain oracle, and cryptocurrency support.

### 2.2. Blockchain-Enabled Dynamic Consent Management

Research on DCMSs remains in its preliminary phase, and very few schemes have been proposed for allowing users to be in control of their data. A flexible and dynamic approach has been developed [11] to capture consent and preserve digital evidence for legal purposes. Emergency cases are considered when users cannot directly provide consent. A framework for evaluating and reporting dynamic consent effectiveness is introduced in [12] to maximize the quality, replicability, and pertinence of decisional autonomy of researcher–participant communication. The EnCoRe project [13] developed schemes to provide reliable and enforceable privacy through dynamic consent capabilities, which enabled data subjects to give and revoke their consent and organizations to enforce it. However, these projects focused on users’ data and privacy enforcement only within an organization. A GDPR-based formal design framework for consent management, using a high-level modeling language to model distributed service-oriented systems was presented in [14]. Most of the previous approaches are centralized solutions, which continue to raise concerns regarding the lack of trusted proof of data provenance, accountability, transparency, security, and privacy. In addition, the opaque nature of such systems provides very limited rights to the users. Furthermore, admins have the authority to manipulate or misuse the granted consents and data without users’ knowledge, making it challenging for users to detect tampering with the consent data. Thus, the TTP remains the single point of failure targeted by attackers to compromise the system. However, blockchain technology enables a decentralized and tamper-resistant trusted data source, and transparency, accountability, and traceability features that can be used to leverage DCMSs.

Genestier et al. [15] discussed the possibilities of using blockchain to manage consent and address privacy and security challenges in the eHealth area. A model of consent management for personal data using blockchain for GDPR compliance is proposed in [16]. Rupasinghe et al. [17] described a privacy-preserving consent model architecture using blockchain to facilitate patient-data acquisition for clinical data analysis. Blockchain-enabled data-sharing consent schemes have been proposed [24,25,26,27] for controlling access to individual health data, where smart contracts are used to denote individual consent and allow requesters to seek and access health data. Table 2 reviews the schemes available in the literature, which are compared with our proposed solution. However, most schemes proposed earlier are domain- and application-dependent, and they do not meet all the requirements previously mentioned.

Our work is different from the schemes previously proposed in the literature. This study is focused on the design and implementation of a dynamic consent management solution using smart contracts to achieve specific, informed, and engaged consent agreements for the legal use of personal data based on GDPR, wherein accountability, transparency, privacy, and security are guaranteed. The permissioned blockchain consortium network is adopted to meet the previously mentioned requirements. These requirements also include secure, confidential, and privacy-preserving interactions and data exchanges among several stakeholders from diverse organizations governed under various policies.

## 3. Proposed System Model

Personal data (GDPR, Article 4) refers to any information related to an identifiable person known as the data subject, whereas data usage refers to any processing operation or set of operations performed on data. These operations include searching, collecting, organizing, adapting, storing, consulting, sharing, and erasing. It is assumed that explicit consent from the data subject is required before collecting and using personal data.

### 3.1. Key Stakeholders and Roles Identification

Based on the GDPR specifications [5], the key stakeholders identified along with their legitimate roles and responsibilities are described as follows:

- Data subject (DS) is a natural or legal person who owns and shares the data while defining privacy and security preferences, and dynamically manages consents (i.e., agree/deny, view, update, and withdraw) to collect and use personal data. Data subjects can also delegate consent rights and audit data collection and usage activity history, so they can withdraw given consent at any time if needed.
- Data controller (DC) is a natural or legal person, public authority, or agency that determines why and how the personal data should be collected and/or used. DC safeguards shared personal data while providing tools for users to dynamically manage consent agreements and control access to their data.
- Data processor (DP) refers to a party (i.e., a natural or legal person, public authority, or agency) that processes personal data on behalf of data controllers. A DP requests for consent and access rights before collecting and/or processing personal data, while recording processing activity history on the blockchain.
- Regulator (RG) denotes the supervisory authorities (i.e., the Office of the Data Protection Commissioner in the European Union) who regulate and control data protection regulations compliance and audit the transaction history to resolve conflicts. The regulator can assign, approve, and revoke membership profile roles.

Each participant’s user role has a set of rights and responsibilities that are well defined and documented.

### 3.2. Consent Requirements and Model Definition

**Figure 1:**
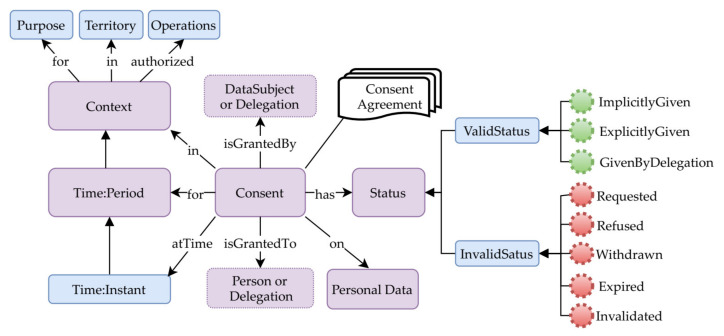
Consent concepts representation using ontology.

Under GDPR (Article 4.11), valid consent should be freely given, specific, informed, and an unambiguous indication that the data subject has consented through a clear agreement statement to the collection and use of his/her data. The core concepts of consent are illustrated in Figure 1 using ontology representation [34]. These include data subject, personal data details, consent requester identity, agreement evidence, context description (i.e., purposes, time, operations, and territory), and consent status, which can be valid or invalid. The consent is granted by the data subject directly or through delegation to a natural person or legal representative. These are essential attributes for legal consent. When personal data collection and use is based on consent, the data controller should be able to prove that the data subject has consented. The consent agreement must specifically contain the consent requester and data controller’s identities, data collection, and usage purposes, as well as the processing activities or operations to be performed. The data subject should have the right to withdraw his or her consent at any time if required (Article 7). Thus, the consent agreement withdrawal should not affect the lawfulness of collecting and using data based on consent before its withdrawal.

**Figure 2:**
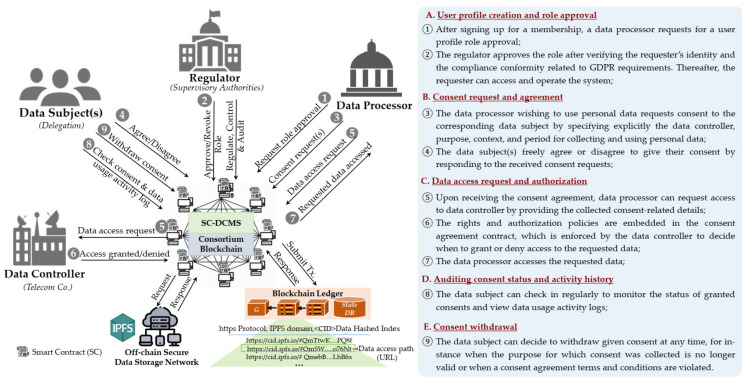
Use cases of the proposed smart-contract-based dynamic consent management system (SC-DCMS) for collecting and using personal data under GDPR.

Figure 2 gives an overview of use cases of the proposed dynamic consent agreement management system for collecting and using personal data under GDPR. Table 3 summarizes the notations used in this paper. Consider a data processor *DP_j_* with *DP* = [*DP*_1_ … *DP_n_*], {*j* | 1 ≤ *j* ≥ *n*}, which might be a data-driven organization (e.g., healthcare organization, research institute, or business company) that wants to obtain consent from a European citizen, that is, data subject *DS_i_* or a set of data subjects formerly registered on a list of members *DS* = [*DS*_1_ … *DS_n_*], {*i* | 1 ≤ *i* ≥ *n*}. The consent request (*CR*) is defined in Equation (1) as a record which consists of a set of the following attributes. *CR_id_* is the consent request identification number. *CA_type_* refers to the type of consent request, which can be classified into three types as defined in Equation (2). Where 0—*default* is for general purpose use cases, 1—*delegated* corresponds to consent delegation cases, and 2—*emergency* denotes the emergency situations when individuals are unable to directly consent on their own.

*CR* = {*CR_id_*, *CA_type_*, *PT_id_*, *RS*, *CXT*, *CST*, *OP*, *AG*, *CR_status_*, *RC_tstamp_*} (1)

(2) $${\mathrm{CA}}_{\mathrm{type}}=\left\{\begin{array}{l} 0,\;\mathrm{default}\\ 1,\;\mathrm{delegated}\\ 2,\;\mathrm{emergency} \end{array}\right.$$

*PT_id_* represents the requested consent agreement participants’ identifiers, which include the data subject, data processor, and data controller identifiers.

*PT_id_* ← {DataSubject (*DS_i_*), DataProcessor (*DP_j_*), DataController (*DC_k_*)} (3)

*RS* denotes the resource or targeted personal dataset identified by *PD_id_* from the relevant *DS_i_* data subject’s personal datasets *PD,* as given in Equation (4).

*RS* ← {*DS_i_*, *PD_id_*}, where *PD_id_* ∈ *PD* = {*PD*_1_,*PD*_2_,…,*PD_n_*} (4)

*CXT* is the consent context specification for collecting and using personal data, which includes the purpose and legal basis of requesting the consent, and the requested time.

*CXT* ← {Purpose (*CR_id_*,*CR_purp_*), LegalBasis (*CR_id_*,*Legal_b_*), RequestedTime (*CR_id_*,*t*)} (5)

*CST* specifies the constraints or conditions under which the consent will be granted. These constraints include the usage period with a specific starting and ending time, and the territory or list of countries to be authorized, as given in Equations (6) and (7).

*CST* ← {*Usage (CR_id_,Prd), Territory (country_list)*} (6)

*Prd* = {*startTime, endTime*} (7)

*OP* indicates the data-processing operations to be performed that are identified by *OP_id_,* which belongs to a set of supported operations. These operations include data searching, collection, storage, processing, disclosing, sharing, and copying.

*OP* ← *OP_id_* ∈ *OP* = {*OP*_1_,*OP*_2_,…,*OP_n_*} (8)

*AG* denotes the agreement status attribute that is defined in Equation (9).

(9) $$\mathrm{AG}=\left\{\begin{array}{l} 0,\;\mathrm{requested}\\ 1,\;\mathrm{agreed}\\ 2,\;\mathrm{denied} \end{array}\right.$$

*CR_status_* denotes the consent request status from the set of attributes specified below.

*CR_status_* = {*Created*,*Confirmed*,*Submitted*,*Agreed*,*Rejected*,*Withdran*,*Closed*} (10)

*CA* refers to the consent agreement that is defined in Equation (11) as a record containing a set of the following attributes. *CA_id_* is the consent agreement identification number. *CR_id_* is the corresponding consent request identifier. *PT_id_* refers to the participants’ identifiers, and *PT_dsign_* to the digital signature of the participants. *RS* indicates the agreed personal dataset list, while *AG* provides the agreement status. *CA _status_* is the consent agreement contract status that can be valid or invalid as given in Equation (11). *CA_tstamp_* is the consent agreement timestamp, and *CR_status_* is the latest consent request status.

*CA* = {*CA_id_, CR_id_, PT_id_, PT_dsign_, RS, AG, CA_status_, CA_tstamp_, CR_status_*} (11)

(12) $${\mathrm{CA}}_{\mathrm{status}}=\left\{\begin{array}{l} 0,\;\mathrm{invalid}\\ 1,\;\;\mathrm{valid} \end{array}\right.$$

Finally, the consent withdrawal (CW) is defined in Equation (13). It is a recording of a set of the following attributes. *CA_id_* is the corresponding consent agreement identifier, and *CWR_id_* is the approved consent withdrawal request identifier. *PT_id_* and *PT_dsign_* refers to the participants’ identifiers and digital signatures, respectively. *CW_rsn_* indicates the consent withdrawal reason, while *CWR_tstamp_* is the withdrawal requested timestamp. *CWR_status_* is the latest status of the consent withdrawal request from the list defined in Equation (14). *CR_status_* indicates the latest status of its corresponding consent request contract.

*CW* = {*CA_id_*, *CWR_id_*, *PT_id_*, *PT_dsign_*, *CW_rsn_*, *CWR_tstamp_*, *CWR_status_*, *CA_status_*, *CR_status_*} (13)

*CWR_status_* = {*Created*,*Confirmed*,*Submitted*,*Approved*,*Rejected*,*Closed*} (14)

### 3.3. System Architecture

**Figure 3:**
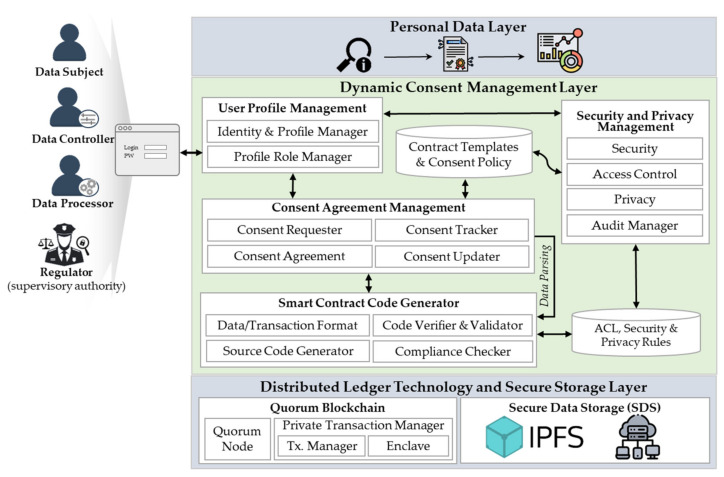
Smart-contract-based dynamic consent management system layered architecture.

The architecture of our proposed system is given in Figure 3. For modularity purposes, the system is divided into three layers: personal data layer, dynamic consent management layer, and DLT and secure storage layer, as described below:

- **Personal Data Layer** provides SC-enabled decentralized applications (*Dapps*) and services for personnel data management, such as data searching, provisioning, and processing features used to perform data analysis. To enable end users to interact with lower layers, Dapps rely on standard Application Program Interfaces (APIs).
- **Dynamic Consent Management Layer** is a middleware layer to feature dynamic consent management using SCs on top of the blockchain. It consists of the following components:
  (1) *User profile management* is in charge of managing the identities, profiles, and roles of participant users. It comprises two sub-components: (a) identity and profile manager, which is in charge of managing the identity and membership profile of participant users; and (b) profile role manager, which manages user role requests, approval, and revocation processes, while mapping user profiles to corresponding roles.
  (2) *Consent agreement management* manages data subjects’ consents throughout the data life cycle. It consists of the following: (a) consent requester, which manages consent requests to collect and use personal data; (b) consent agreement, which allows data subjects to systematically provide and manage varied types of consent agreements on each requested personal dataset; (c) consent tracker, which traces and tracks the consent transaction logs stored on the blockchain ledger, making all stakeholders accountable for their activities; (d) consent updater, which enables data subjects to update and adapt their consent agreement preferences (i.e., withdraw or revoke consent) based on the evolving context.
  (3) *Smart contract code generator* produces SCs based on predefined contract templates and consent agreement policies. It is composed of the following: (a) data/transaction format checks for provisioned data and transaction formats’ mutual compatibility; (b) source code generator creates the consent-agreements-related SCs to ensure contractual reliability in a machine-readable source code; (c) code verifier and validator checks for compatibility, correctness, and validity of the generated SCs to ensure that they can operate without errors and security vulnerabilities; and (d) compliance checker verifies privacy and security policies against legal compliance before the SC is published and deployed on the blockchain.
  (4) *Security and privacy management* comprises: (a) security manager, which provides data security-related features, like authentication, authorization, and confidentiality; (b) access control manager, which authorizes or denies access to personal data depending on access control policies and rules embedded in consent agreement contracts; (c) privacy manager, which helps users define and manage their privacy preferences; and (d) audit manager, which enables users to audit the history in terms of who requested (and granted) access to their data, when the data were used, and by whom. The user profile, security, and privacy management components extend the generic permissioned features provided by the lower layer.
- **Distributed Ledger Technology and secure storage layer** provides (1) a Quorum-blockchain-based [16] immutable transaction shared ledger and state database, maintained by a consensus of peer nodes in a consortium blockchain network, and (2) a P2P secure data storage system. This layer provides an operating environment for running SCs that enforces the consent agreement requirements.
  (1) *Quorum blockchain* [16] comprises two core sub-components: (a) a Quorum node, which is a lightweight forked version of the go-Ethereum client (known as geth) that was modified for supporting contract and transaction privacy, and (b) a private transaction manager *(PTM)* module that is divided into two sub-modules, namely the transaction manager *(TM)* and enclave. The *TM* manages private transactions by allowing access to encrypted transaction data and exchanging encrypted payloads between participant nodes. The enclave is a distributed ledger protocol that provides cryptographic methods for participants’ authentication, transaction authenticity, and historical data security. It works with the TM to leverage privacy by managing the symmetric key generation, data encryption, and decryption independently.
  (2) *Secure data storage (SDS)* orchestrates the data storage and access in a distributed storage system. SDS nodes store off-chain all the shared personal data and consent agreement forms in a peer-to-peer data storage network using IPFS protocol [15]. The hashed indexes of the data are recorded on a blockchain ledger and access is controlled using SCs. These nodes ensure the reliability, accessibility, and integrity of the data.

This layer also provides a *blockchain oracle service* (BOS) that leverages SCs embedded within Dapps to enable secure data exchange between the blockchain and the outside world (off-chain). SC-DCMS relies on the Quorum TM to guarantee a trusted on-chain and off-chain data exchange between the stakeholders.

### 3.4. User Profile and Personal Data Management

In this subsection, we describe the approach used to create and approve user profiles. Then, we look at how personal data is managed using the proposed system.

#### 3.4.1. User Profile Creation and Role Approval

A given user profile may be assigned one or multiple roles. For instance, a user profile may have controller and processor roles if the organization has both roles. The membership user profile creation, role request, and approval steps are as follows:

(1) Sign up for a membership user account of the consortium blockchain network, which results in the creation of a user profile;
(2) Use the user-profile-associated information to request approval for the specific role(s) in the system;
(3) The role approval requests are approved by the regulator(s) after verifying the identities of the requesters and the regulatory compliance requirements;
(4) Upon receiving the role approval, the user profile status becomes active to use and manage in the system.

#### 3.4.2. Personal Data Management

The personal data used includes medical facility visit records, vital signs, laboratory test results, contact location, movement based on the Global Positioning System (GPS), and financial transaction records [3]. These data are generated by several organizations (e.g., banks, hospitals, telecom companies, public service offices, and research institutes) and can be exchanged securely among involved entities using a permissioned blockchain network. The proposed system provides a mechanism for creating and sharing personal dataset profiles on the blockchain. First, data subjects or third-party organizations create the dataset profiles. The dataset profiles contain hashed indexes connected to the data. Second, the dataset profile publication requests are sent to be approved by peer data controllers. Later, the dataset profiles are published to the blockchain, and, upon successful execution, their details are sent to the data subjects. For privacy and confidentiality purposes, personal data are assumed to be anonymized and encrypted before being shared on blockchain using hashed indexes of the datasets stored off-chain in SDS nodes.

### 3.5. Smart-Contract-Based Dynamic Consent Management

This subsection provides details related to the SC-DCM operations that comprise consent expression, consent request, and agreement, as well as consent withdrawal.

#### 3.5.1. Consent Expression

Users can express their consent in a variety of ways, such as by filling in a form or ticking a box on a web page. The consent and rights expression model is defined using the eXtensible Access Control Markup Language (XACML) [35]. Listing 1 provides an example of consent expression and rights definition. To standardize and simplify integration with the Policy Enforcement Points (PEP) and Policy Decision Points (PDP), consent and rights are defined using a JSON profile of XACML [36]. This is then converted into XACML policies used by role-based access control systems [35]. The security and privacy management module functions as the policy enforcement point of SC-DCMS.

Listing 1 **Consent expression and rights definition example in JSON.**

1. Consent_Agreements {

2.   Consent_Agreement [

3.   { ”AgreementNum”: ”202102-0001021410”,

4.    ”Type”: ”0”, // 0-Default, 1-Delegated, 2-Emergency

5.    ”Participants”: [

6.     {”by”: ”DataSubjectID”,”to”: ”DataProcessorID”, ”controller”:”DataControllerID”} ],

7.    ”Resource”: [ ”https://cid.ipfs.io/#QmTwK…PQ8f”, ” https://cid.ipfs.io/#QmS….6Nt” ],

8.    ”Context”: [

9.      { ”for”: ”Public Health Emergency”,

10.      ”at”: ”2021-02-10 T13:30:10”,

11.       ”legal”: ”GDPR, Article 4.11” } ],

12.   ”Constraints”: [

13.      { ”from”:”2021-02-10”,”until”: ”2021-03-30”,

14.      ”in”: [”EU”, ”South Korea” ] } ],

15.    ”Operations”: [”COLLECT”,”STORE”,”PROCESS”,”DISCLOSE”,”COPY”,”SHARE” ],

16.    ”Agreement”: ”ALLOWED”,

17.    ”Status”: ”VALID”

18.   } ]

19. }

#### 3.5.2. Consent Request and Agreement

**Figure 4:**
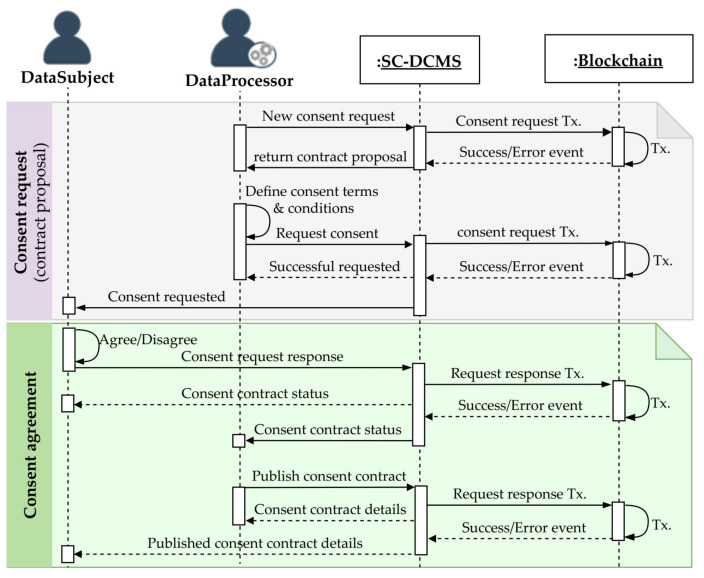
Consent request and agreement between participating entities of the SC-DCMS.

Figure 4 describes the consent request and agreement processes between two participants of the SC-DCMS. A data processor as consent requester is seeking consent on a particular personal information dataset by sending a consent request, which is processed by the SC-DCMS and recorded on the blockchain, after which the data subject is notified upon successful execution. The consent request includes the data subject and consent requester identifiers, purposes, period, and legal basis to collect and/or use personal data. Upon receiving the consent request, the data subject freely decides to agree or disagree by sending a request-response to the requester. The consent agreement process ends with the creation of a consent agreement contract published on blockchain upon successful execution of the transaction. The detailed consent request algorithm is given in Algorithm 1, whereas Algorithm 2 describes the proposed consent agreement algorithm.
**Algorithm 1** Consent agreement request
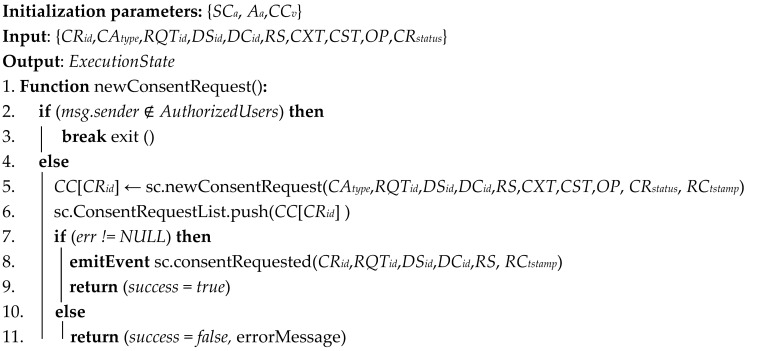


**Algorithm 2** Consent agreement

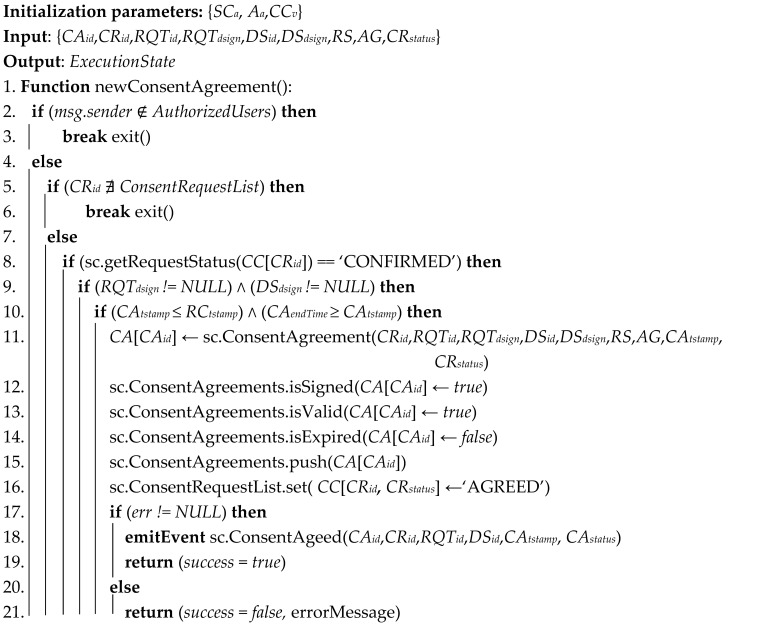



#### 3.5.3. Consent Withdrawal

**Figure 5:**
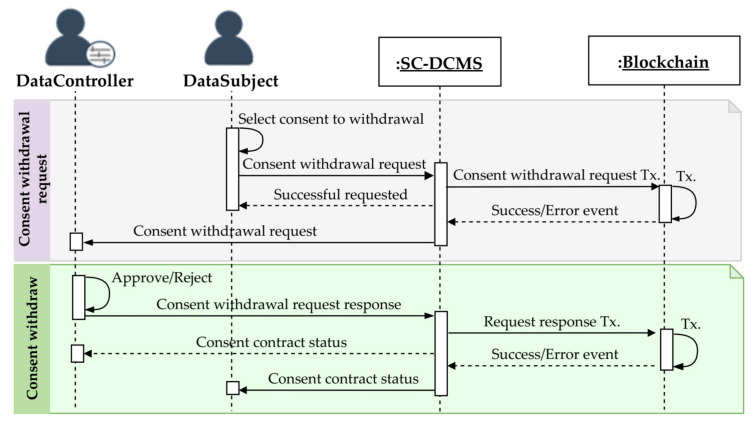
Consent withdrawal sequence diagram of the SC-DCMS.

The data subject can withdraw given consent at any time, for example, when the reason for which the consent was collected is no longer valid or when the agreement clauses are violated. Figure 5 illustrates the consent withdrawal mechanism processes of the SC-DCMS. The data subject starts by selecting the consent to be withdrawn and submits a withdrawal request for approval to the corresponding data controller(s). After getting approved, the targeted consent agreement contract is withdrawn by changing its status as invalid upon successful execution of the transaction. To exercise their rights to be forgotten, using smart contracts, users can request deletion of their data stored off-chain, but the record of these transactions is retained in the blockchain ledger. The detailed consent agreement withdrawal algorithm is given in Algorithm 3.
**Algorithm 3** Consent withdrawal
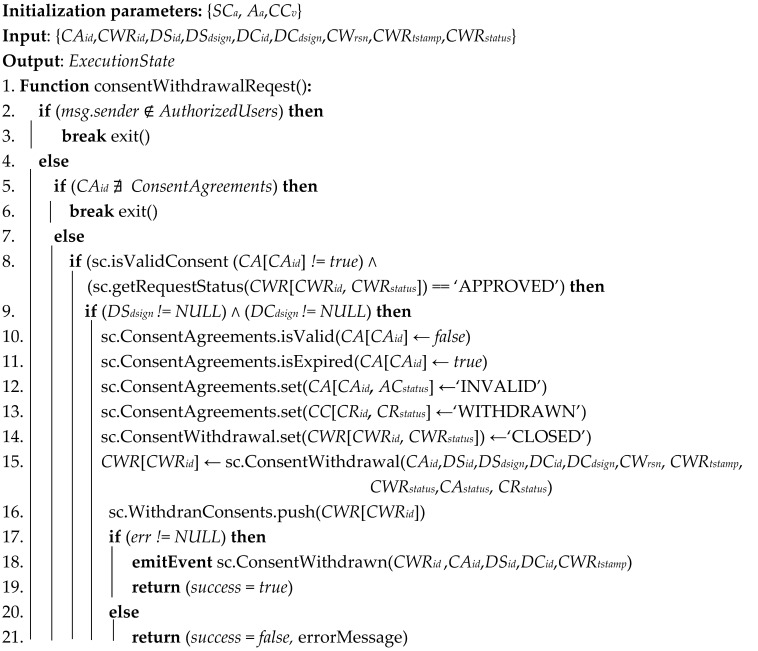


Events are transaction receipts that contain log entries providing information about actions taking place during the execution of transactions. Emitted events are used to store the arguments passed in transaction logs stored on the blockchain. Thus, the consent and data usage activity history can be easily traced and tracked using event logs.

## 4. Implementation and Experiments

This section elaborates on the consent contract generation mechanism, its implementation, and experimental details of our proposed system.

### 4.1. Consent Contract Generation

The consent agreement SC source code is generated by the SC-DCMS from a consent agreement form presented in a human/machine-readable format using XML/XHTML-like forms. The current implementation of SC-DCMS provides support for SCs written in Solidity. To guarantee that the generated SCs are secure and trusted, the SC-DCMS checks first for compatibility, correctness, and validity to ensure that they will run without errors and security vulnerabilities. The privacy and security policies are verified against predefined legal compliance rules before SCs are deployed on the blockchain.

### 4.2. Implementation Details

**Figure 6:**
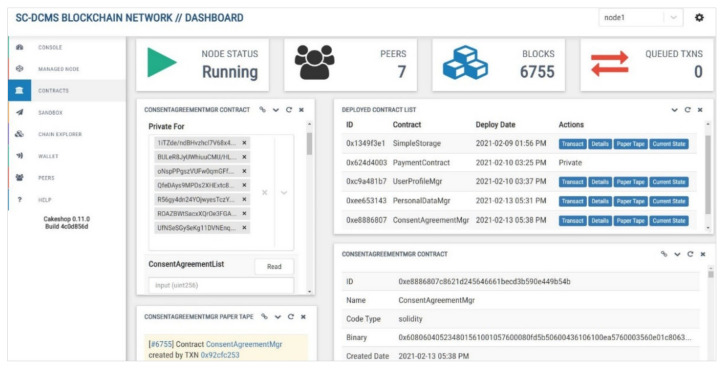
SC-DCMS consortium blockchain network management dashboard using Cakeshop.

For the implementation of the prototype of our system, we have adopted GoQuorum blockchain [16], which is an Ethereum-based permissioned blockchain platform with advanced enterprise-grade features enabling contract and transaction privacy, pluggable consensus protocols, and scalable performance. Cakeshop sandbox [37], an integrated development environment and software development kit, was used to develop, compile, and deploy our SCs written in Solidity language. JSON-RPC, Web3, and REST APIs are used to interact with the SCs and blockchain. To test and validate the correctness of our SC-DCMS, the blockchain infrastructure was deployed on premise in a Docker container environment. The experimental setup environment is described in Table 4. Docker Compose configuration files were used to generate a consortium blockchain network, composed of seven peer nodes with their respective transaction managers and ethloggers. Each node had a digital wallet that contained the user’s credentials, keys, account addresses, and balances. Tessera [38] was integrated as a transaction manager to encrypt, decrypt, and distribute private transactions in the Quorum blockchain network using Constellation [39], a self-managing and peer-to-peer communication system for secure messaging with clients and other nodes. IBFT [40] and RAFT [41] consensus protocols were adopted for faster consensus and immediate transaction finality. Cakeshop [37] was used to explore, monitor, and manage all the nodes and resources of the consortium blockchain network, as shown in Figure 6. Cadvisor [42] was set up to monitor containers’ resource usage and performance metrics. A Quorum reporting server [43] collected the blockchain network metrics, whereas a Splunk App for Quorum [44] monitored in real time the health of our deployed blockchain infrastructure. IPFS [15] nodes were used as secure decentralized storage to store the consent agreement forms, contract templates, and personal datasets off-chain.

## 5. Evaluation

In this section, we analyze the security and privacy of our SC-DCMS and assess its features to ensure that it meets the design requirements for acceptability and reliability. Finally, we evaluate the performance of the system.

### 5.1. Security and Privacy Analysis

The security and privacy of SC-DCMS rely on and extend advanced features of the underlying Quorum permissioned blockchain. The implemented code was successfully analyzed and verified using highly precise vulnerability verification tools for Solidity SCs, namely VeriSmart [45] and SmartCheck [46]. These SCs are secure against currently well-known vulnerabilities [45,46], such as reentrancy, integer underflows, and overflows, access control, denial of service (DoS), and timestamp manipulation.

As Quorum has removed the transaction gas fees paid in the Ethereum public blockchain for the computational effort needed to complete transactions [47], issues of running out of gas while using our system no longer occur, because in Quorum, a set of validator nodes is defined to secure the network. All members of the consortium network are assumed to behave in a trustworthy manner. Each organization has a set of roles and nodes defined with a set of network access control permissions. The RAFT consensus protocol provides a crash fault tolerance support for system and network availability. RAFT requires 2*f* + 1 nodes to be set up in a network to tolerate *f* faulty nodes [41], whereas IBFT offers a Byzantine fault tolerance mechanism and can tolerate *f* number of faulty nodes in a network of *n* = 3*f* + 1 nodes [40]. Data integrity protection is achieved via the blockchain, which has all transaction data hashed and timestamped. To ensure that our system design satisfies fundamental data protection regulatory requirements, a GDPR-based framework was used for data protection impact assessment (DPAI) [48].

Private contracts and transactions are supported by Quorum through a separation of public and private states while using P2P encrypted message exchanges to directly transfer private data between network participants [16]. Furthermore, to preserve privacy and ensure that participants’ identities remain hidden, the transaction data are replaced by encrypted hashes. Personal data are anonymized and encrypted before being shared on the blockchain by using hashed indexes of the datasets stored off-chain in SDS nodes. In the interests of confidentiality, user profiles of the participants have separate roles to enable role-based access control management. The participants are required to sign up for a membership profile and get authorized before joining and using the system. They can define custom consent security and privacy policies on their data and control who can collect, access, and use their data shared on blockchain. All data-related transaction records are preserved on the blockchain as tamper-proof evidence to guarantee trusted data provenance, transparency, and accountability. Thus, SC-DCMS enables data controllers to easily perform privacy impact assessments (PIAs) [49] and/or DPAIs [48] and document them before starting any data-processing activity for legal compliance under the GDPR.

### 5.2. Satisfying Design Requirements

After performing several validation tests for system acceptability and reliability, we have confirmed that the features of the proposed system fulfill the aforesaid DR1–DR6 derived from GDPR specifications (Section 1.1), as summarized below:

- *Key stakeholders and roles identification*: Four key stakeholder participants were identified to fulfill DR1. Their legitimate roles and responsibilities are described in Section 3.1. Each participant user must sign up for a membership user profile and get authorized before joining the network and using the system.
- *User-centric dynamic consent management*: DR2 is satisfied by our proposed user-centric and paperless consent management system enabled by smart contracts. It enables data subjects to dynamically control their consent on the personal data collection and usage over its lifecycle.
- *Consents and rights expression*: Individual consents, and related rights are systematically and explicitly expressed in a human/machine-readable format to satisfy DR3. Furthermore, it allows users to delegate their consent rights if required.
- *Consent and data usage activity history accessibility*: The blockchain-based shared immutable leger is used for traceability and accountability of consents and data usage transaction records. Thus, data subjects can easily view and audit data collection and usage activity history to satisfy DR4.
- *Consent violation notification*: To fulfil DR5, our system provides consent agreement terms violation detection, notification, and reporting mechanisms using SCs.
- *Consent agreement withdrawal*: The proposed consent withdrawal mechanism satisfies DR6 (Article 7.3) by providing users the possibility to withdraw a given consent agreement when the purpose for which the personal data was collected is no longer valid or the consent agreement terms are violated.

Consequently, data controllers can rely on SC-DCMS to provide trusted and reliable consent management services enabling users to manage consent over their data.

### 5.3. Performance Evaluation

The system performance is evaluated in terms of complexity of algorithms, transaction throughput and latency, computing resource consumption, and storage network bandwidth utilization. The experiments were conducted to investigate how the transaction workload variations could affect the system performance with RAFT and IBFT consensus algorithms.

**Figure 7:**
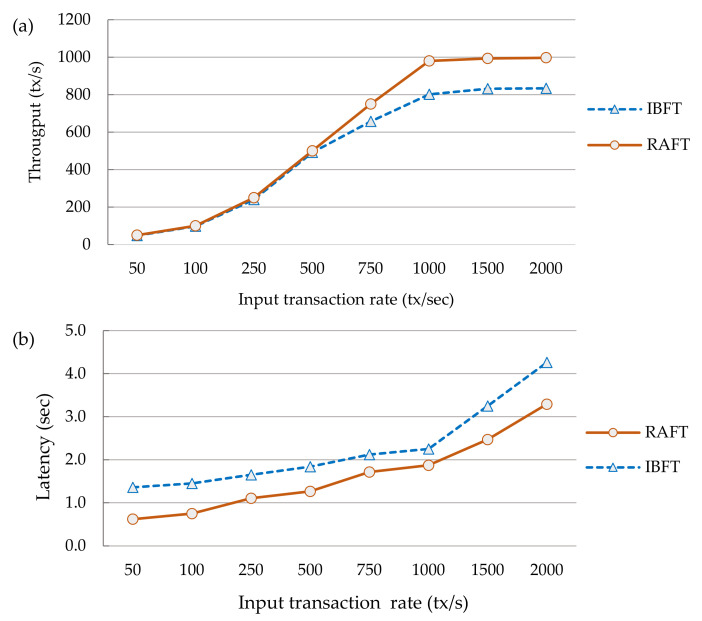
(**a**) Transaction throughput evaluation of IBFT vs. RAFT and (**b**) transaction latency evaluation of IBFT vs. RAFT.

**Figure 8:**
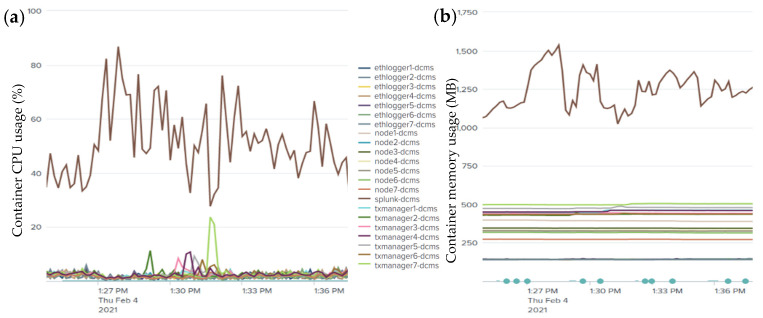
System computing resource usage: (**a**) container system CPU usage (%) and (**b**) container memory usage (MB).

**Figure 9:**
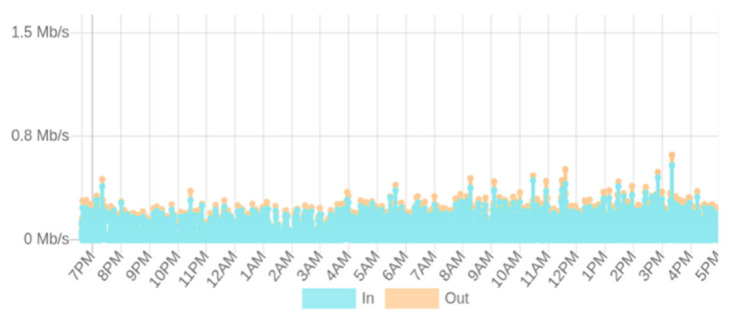
Network bandwidth utilization of the IPFS storage system.

(1) *Computation time and space complexity*: The complexity of our proposed algorithms is assessed to determine their efficiency in terms of computation time and space consumption. Considering Table 5, the complexity of a newUserProfile creation and roleApproval transaction is *O(1)*, which means only a single read-write operation is required to verify if a given record does not exist before writing it to the blockchain. The computation complexity of addNewData and newConsentRequest transactions is also *O(1)*, whereas the complexity of newConsentAgreement, newConsentWithdrawalRequest, and consentWithdrawal transaction is *O(2),* indicating that two read-write operations are required. In general, the first read operation is to check if the given record already exists in the ledger to avoid duplication, and the second is to collect required information to perform the transaction. For write operations, the first is to write the pushed transaction output data to the blockchain and the second is to update the status of related records in the ledger. The complexity of a smart contract algorithm affects the transaction execution time and latency. The evaluation has revealed that our proposed algorithms have a linear time complexity, which increases linearly with the size of input transactions. Table 6 provides the time and space complexity of a transaction and block in average values. For simplicity, the number of transactions per block is set by default to one. The space complexity of major SCs of our system is given in Table 7.
(2) *Effect of input transaction rate variation on the transaction throughput and latency*: The transaction throughput refers to the number of transactions processed per second (TPS) by the blockchain network [47,50], which is computed using Equation (1):
  (15) $${\mathrm{Tx}}_{\mathrm{throughput}}=\frac{\Sigma_{\;i=1}^{\;n}{\mathrm{Tx}}_{\mathrm{succ}}\;}{\Sigma_{j=1}^{\;n}{(\mathrm{Comp}}_{t}{-\;\mathrm{EX}}_{t})\;}\;$$
  where ${\mathrm{Tx}}_{\mathrm{succ}}$ denotes successfully executed transactions, *n* is the number of input transactions, ${\mathrm{EX}}_{t\;}$ refers to the transaction execution time, and ${\mathrm{Comp}}_{t}$ is the transaction completion time. The transaction latency is the time elapsed between the transaction submission and the response reception—after the transaction is successfully included in a block, committed, and confirmed on the blockchain [47,50]. Equation (2) is used to calculate the transaction latency.
  (16) $${\mathrm{Tx}}_{\mathrm{latency}}=\frac{\Sigma_{j=1}^{\;n}{\mathrm{Comp}}_{t}\;}{\Sigma_{i=1}^{\;n}{\mathrm{Tx}}_{\mathrm{succ}}}$$
  To understand the impact of the input transaction rate variation on the transaction throughput and latency, in our experiment, we generated a variable workload ranging from 50 to 2000 tx/s input transaction rates to stress-test the system. As read operations do not change the state of the ledger, our focus was on write transaction workloads that randomly update a selected key-value store of the deployed private contracts. The private transaction event generator scripts were launched from a client console to be broadcast to all the peer nodes. The experiment was repeated for three rounds for the Quorum blockchain network running with IBFT and RAFT consensus protocols. Later, we calculated the average transaction throughput and latencies. The plots in Figure 7a,b respectively depict the comparison between the IBFT and RAFT transaction throughput and latency. We observed that the throughput was scaling linearly for low transaction submission rates up to 802 and 980 tx/s for the IBFT and RAFT consensus, respectively. Beyond this, the throughput did not increase much until it reached a maximum point of 834 tx/s for IBFT and 1000 tx/s for RAFT, and then started to generate a lot of errors. In contrast, both consensus algorithms showed that the latency scaled linearly for all the input transaction rates. As can be seen, RAFT slightly over-performs IBFT.
(3) *Effect of input transaction rate variation on the computing resource consumption*: We monitored in real time the system performance to evaluate the impact of input transaction rate variations on the CPU and memory of the system utilization by the containers deployed in our infrastructure, as shown in Figure 8a,b. It can be seen that the system has a moderate resource consumption, requiring less than 500 MB of memory and 10% of CPU utilization on average to keep the nodes running. However, the Splunk server container (SSC) used more resources as compared to the others. This is because it was monitoring and reporting the health state and metrics of the deployed infrastructure every 3 to 5 s continuously. We observed that the resource usage scaled with the variations in the transaction workload. SSC reached a peak of 87% and 1525 MB whereas one of the remaining nodes went up by 23% and 512 MB of the average CPU and memory usage, respectively.
(4) *Effect of input transaction rate variation on the storage network bandwidth consumption*: The bandwidth utilization was monitored for the IPFS storage network traffic to investigate the impact of the transaction workload variations on the bandwidth usage. Figure 9 gives a summary of the input and output network bandwidth utilization of the IPFS-based storage system.

The results of our experiment revealed that a minimum of 409.6 Kb/s of bandwidth was required on average to keep the IPFS nodes running. This has optimized the overall performance of SC-DCMS for the IBFT and RAFT consensus algorithms, reaching a peak of 834 and 1000 tx/s throughputs with 4.26 and 3.32 s latencies for a maximum of 2000 tx/s input transaction rates for IBFT and RAFT, respectively. As compared to IBFT, the RAFT consensus performed better with less resource consumption. This is owing to its faster block times (with a minimum of 1 ms), transaction finality, and on-demand block creation. The IBFT algorithm minted blocks at a constant rate every 1 s, and even empty blocks were minted, which used excessive storage and created a lot of messaging overheads.

## 6. Limitations and Open Challenges

In this section, we elaborate on some limitations and open challenges that need further research.

- *System complexity and key management*: The complexity of our proposed solution makes the key management very challenging, as it is integrated with several systems and platforms. Efficient and user-friendly key management schemes are required to take advantage of the permissioned blockchain-enabled security and privacy features.
- *Oracle problem with smart contracts and blockchain*: Data exchange between the outside world and blockchain is handled by SCs integrated with Dapps. This may lead to an oracle problem if it is not correctly implemented [51,52].
- *GDPR and immutable nature of blockchain*: Withdrawing consent under GDPR might require, in some cases, deleting stored personal data for users to exercise their rights to be forgotten (Article 17). However, this remains a complex and challenging issue because of the immutable nature of blockchain wherein further research is required to be effectively applied and implemented [53,54].
- *Smart contract vulnerabilities*: Developing completely correct and bug-free smart contracts is very challenging. As SCs are gain in popularity, this will raise new security issues and challenges. Efficient smart contract vulnerability and security auditing solutions are very critical.
- *Smart contract adaptability and upgradability*: As SCs are immutably stored on a blockchain once deployed, they cannot be updated or upgraded for patching bugs or security vulnerabilities. This becomes a major challenge when adapting to evolving privacy, security, and legal compliance policies.
- *Automated security and privacy policies verification and GDPR compliance checking*: There is still an urgent need for user-friendly audit engines to automatically verify security, privacy, and legal compliance policies more efficiently.

## 7. Conclusions and Future Work

We have proposed a user-centric dynamic consent management system using smart contracts for lawful personal data usage under GDPR. Our system design is aligned with the GDPR requirements. It empowers individuals to systematically provide various types of consent agreements on distinct personal information and manage their consented data usage. IPFS-protocol-enabled decentralized storage nodes were integrated with our system to store data off-chain. Blockchain technology was used to enforce trust between participants and for immutable data transaction history and access log recording, trusted data provenance, and accountability features. Users can delegate their consent rights and audit them by tracking the event logs on the blockchain. Users are notified about any consent violation and can withdraw consent at any time. We designed and implemented a prototype of the proposed system, which is integrated with GoQuorum blockchain to demonstrate the feasibility of our concept. The implemented SC code and artifacts are publicly available on GitHub, with a description of how to reproduce the test results. Experimental testing and validation processes were conducted to assess its acceptability and reliability. The settings of our system can be easily adapted and generalized to support a wide range of industries and use cases. We also assessed the system’s security and privacy as well as its performance in terms of SC algorithm computation time and space complexity, transaction throughput and latency, computing resources, and storage network bandwidth usages. The experiments were conducted to examine the impact of the transaction workload variation on the overall system performance with RAFT and IBFT consensus protocols. The experimental results showed that the proposed system achieved high transaction throughputs and low latencies with moderate resource consumption and storage network bandwidth utilization.

Above all, this study provides useful theoretical and practical background for further research to be carried out in the future. Future directions to investigate are as follows:

- The experiments in this study were performed on a single server with nodes running in containers. In the future, we plan to consider using a cluster or cloud computing services to deploy our solution and conduct in-depth performance and scalability analyses for a variable number of nodes and batch sizes.
- We plan to design an automated compliance-checking engine based on the techniques described in other studies [55,56] for compliance with data protection regulations and verification of security policies.
- Novel efficient and user-friendly key management approaches are worthy of further investigation.
- Future research can try to develop systematic auditable and privacy-preserving proof of compliance schemes for consent revocations that require personal data deletion by data controllers.

## Figures and Tables

**Table 1:** Comparison of the five popular blockchain platforms: Ethereum, Hyperledger, R3 Corda, Ripple, and Quorum.

Features	Ethereum ^1^	Hyperledger Fabric ^2^	R3 Corda ^3^	Ripple ^4^	Quorum ^5^
Targeted industry	Cross-industry	Cross-industry	Financial	Financial	Cross-industry
Mode of operation (ledger)	Permissionless (public)	Permissioned (private)	Permissioned (private)	Permissionless (public)	Permissioned (public/private)
Consortium network support	X	√	√	X	√
Decentralization	Decentralized	Partially	Partially	Decentralized	Decentralized
Transaction/smart contract privacy	X/X	√/√	√/√	X/X	√/√
Consensus protocols	PoW/PoS	Pluggable	Notary-based	Probabilistic voting	Pluggable
Transaction throughput	~20 tps	>2000 tps	~170 tps	~1500 tps	~1000 tps
Smart contract support	√	√	√	√	√
Blockchain oracle	√	√	√	√	√
Cryptocurrency	ETH	N/A	N/A	XRP	ETH

^1^https://ethereum.org/ (accessed on 25 November 2021); ^2^ https://github.com/hyperledger/fabric (accessed on 25 November 2021); ^3^ https://www.corda.net/ (accessed on 25 November 2021); ^4^ https://ripple.com/; ^5^ https://consensys.net/quorum/ (accessed on 25 November 2021).

**Table 2:** Comparison of the proposed system with related works.

Paper	Legal Basis	User-Centric	Consent Agreement/Delegation	Auditability/ Withdrawal	Privacy/Security	SC Language	Blockchain Platform	Type of Network	Use of IPFS ^3^	Implementation	Performance Evaluation
[11]	DPD ^1^	√	√/√	X/√	X/X	X	X	X	X	X	X
[12]	-	√	√/X	-	-	X	X	X	X	X	X
[13]	-	√	√/X	X/√	√/√	X	X	X	X	√	X
[14]	GDPR	√	√/X	√/X	√/√	X	X	X	X	X	X
[15]	-	√	√/X	X/√	-	-	HLF ^2^	Private	X	Prototype	X
[16]	GDPR	√	√/X	-	X	X	-	-	X	X	X
[17]	GDPR	√	√/X	√/X	-	-	-	-	X	X	X
[24]	-	√	√/X	√/X	X/√	Solidity	Ethereum	Public	X	Prototype	√
[25,26]	-	√	√/X	√/X	√/√	Solidity	Ethereum	Public	√	Prototype	√
[27]	GDPR	√	√/X	√/√	X/√	JavaScript	HLF	Private	X	√	X
[28]	GDPR	√	√/X	√/√	√/√	JavaScript	HLF	Private	X	Prototype	X
[29]	-	√	√/X	√/√	√/√	Go	HLF	Private	X	Prototype	X
[30,31]	GDPR	√	√/X	√/X	X/√	Solidity	Ethereum	Public	X	Prototype	X
[32]	GDPR	√	√/X	√/√	X/√	Go	HLF	Private	X	Prototype	√
[33]	X	X	X/X	X/X	X/√	Solidity	Ethereum	Public	X	Prototype	√
Our work	GDPR	√	√/√	√/√	√/√	Solidity	Quorum	Consortium	√	Prototype	√

^1^ The EU Data Protection Directive (DPD); ^2^ HLF: Hyperledger Fabric; ^3^ IPFS: InterPlanetary File System.

**Table 3:** Notation description.

Notation	Description
*DS*, *DC*, *DP*, *RG*	data subject, data controller, data processor, regulator
*RQT*, *PT*	requester, participants
*CC*, *CR*, *RC*	consent contract, consent request, request creation
*RS*, *PD*, *OP*	resource, personal data, operation
*CXT*, *Legal_b_*, *Prd*	context, legal basis, period of usage
*CST*, *Territory*	constraint, territory of use
*A*, *_a_*, *AG*, *CA*	account, address, agreement, consent agreement
*CW*, *CWR*, *SC*, *_v_*	consent withdrawal, consent withdrawal request, smart contract, version
_*id*, *dsign*, *status*, *purp*, *rsn*_	identifier, digital signature, status, purpose of use, reason
_*tstamp*, *startTime*, *endTime*_	timestamp, starting time, ending time
*OS*, *IPFS*, *EVM*	operating system, interplanetary file system, Ethereum virtual machine

**Table 4:** Experiment environment setup.

**Hardware**	**Description**
CPU	AMD^®^ Ryzen 7 1700-8 Core
GPU/RAM/SSD	NV132/64 GB/2 TB
Network interface	I211 Gigabit Network
**Software**	**Description**
OS	Ubuntu 20.04.2 LTS, 64bit
Network generation	Docker-compose v1.25.0
Quorum version	Quorum 20.10.0
Consensus protocol	IBFT, RAFT
Number of nodes	7
Splunk Enterprise Server	v8.0.4
Splunk App for Quorum	v1.0.9
Client	Geth/node-raft/v1.9.7(quorum-v20.10.0)/linux-amd64/go1.13.15
Nodejs/Npm/Cakeshop	v10.19.0/v6.14.4/v0.11.0
Solidity compiler EVM	Constantinople
IPFS	go-ipfs v0.7.0

**Table 5:** Computation complexity of essential transactions interacting with the blockchain.

Type of Transaction	*R* ^1^	*W* ^2^
newUserProfile	*O(1)*	*O(1)*
roleApproval	*O(1)*	*O(1)*
addNewData	*O(1)*	*O(1)*
newConsentRequest	*O(1)*	*O(1)*
newConsentAgreement	*O(2)*	*O(2)*
newConsentWithdrawalRequest	*O(2)*	*O(2)*
consentWithdrawal	*O(2)*	*O(2)*

^1^ *R*: read operation, ^2^ *W*: write operation.

**Table 6:** Time and space complexity of a transaction and block.

Parameter	IBFT	RAFT
Transaction latency (sec)	0.0018	0.0015
Transaction size (KB)	2.380	2.378
Block size (KB)	4.247	4.245
Number of transactions per block	1	1
Default block time (sec)	1	0.05

**Table 7:** Space complexity of core smart contracts.

Smart Contract	Value
PersonalDataMgr (KB)	10.149
UserProfileMgr (KB)	12.438
ConsentAgreementMgr (KB)	18.117
